# Drug Coverage Policy and Legacy Prescribing: A Cross‐Sectional Analysis in British Columbia

**DOI:** 10.1002/hsr2.71718

**Published:** 2026-01-27

**Authors:** Aydan Con, Ivy Thrasher, Aaron M. Tejani

**Affiliations:** ^1^ Therapeutics Initiative, Department of Anaesthesiology, Pharmacology & Therapeutics, Faculty of Medicine University of British Columbia British Columbia Canada

## 1 Introduction

Legacy drug‐prescribing, the practice of continuing fixed‐term drug therapy indefinitely without strong evidence of long‐term therapeutic benefit, is a growing concern in healthcare [1]. Legacy drug prescribing can contribute to polypharmacy, which is associated with adverse health outcomes [2]. Several studies have highlighted significant proportions of elderly patients receiving legacy drug prescriptions for antidepressants, bisphosphonates, and proton‐pump inhibitors (PPIs) [1, 3, 4]. In British Columbia, the provincial government operates the BC Pharmacare Drug Plan, which provides medication funding for individuals [5]. Within this plan, the Limited Coverage program (BCPLC) funds medications only when patients meet specific medical criteria, with coverage typically granted for defined durations [5].

Because such funding rules shape access, drug policy itself can influence prescribing patterns. Irish data show polypharmacy is more common among patients with subsidized coverage than those paying out‐of‐pocket [4]. Medicare Part D similarly increased both essential and low‐value drug use [3], while British Columbia's reference pricing policy shifted patients to lower‐cost equivalents without harm [6]. Together, this evidence suggests that funding arrangements can either reinforce problematic polypharmacy or promote more cost‐effective prescribing. This study aims to assess whether BCPLC funding criteria contribute to legacy drug prescribing specifically and/or problematic polypharmacy.

## 2 Methods

We analyzed the 2022 BCPLC formulary, comprising 302 medications, focusing on the covered indications and duration of therapy (DOT) for each medication [5]. A multidisciplinary research group consisting of two pharmacist members of the BC Ministry of Health, a pharmacist and two research assistants developed a standardized data collection form. This form extracted the BCPLC covered indications and funding durations, as well as the listed indications and durations found in best‐available evidence, such as systematic reviews, Health Canada drug monographs, Canadian Agency for Drugs and Technologies in Health (now known as Canada's Drug Agency) reimbursement recommendations, and clinical databases like UpToDate and Lexicomp [7]. We compared BCPLC criteria with referenced durations and indications and identified two types of mismatches: duration of therapy mismatch (DOTM) and indication mismatches (IM) [5]. We looked to see if medications with mismatches were listed as potentially inappropriate according to Beer's 2019 criteria [8]. All analyses were descriptive only; no statistical calculations were done. No Research Ethics Board approval was required as this was a quality assurance project.

## 3 Results

**Figure 1:**
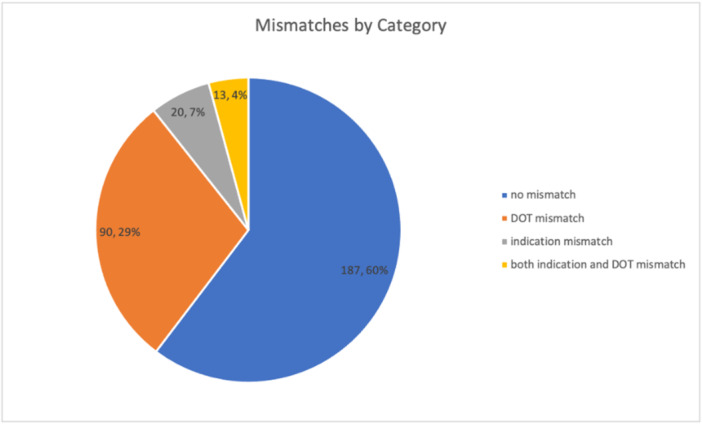
The proportion of BCPLC list with no mismatch (*n* = 187), DOTM (*n* = 90), IM (*n* = 20), and both IM and DOTM (*n* = 13).

Of 286 medications analyzed, 40% exhibited at least one mismatch (see Figure 1), predominantly DOTMs, largely comprised of NSAIDs, PPIs, and bisphosphonates [5].

**Figure 2:**
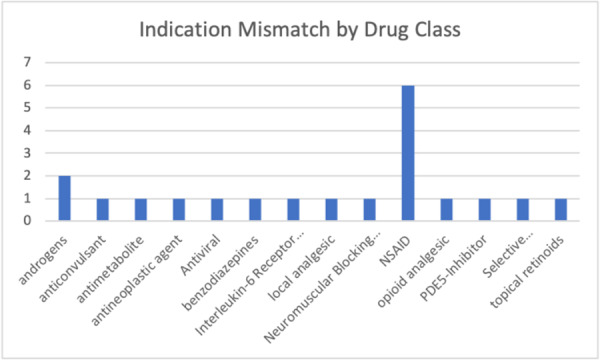
Indication mismatches by therapeutic class.

We identified four subcategories of DOTMs (see Figure 2, *n* = 90): (a) medications with indefinite BCPLC coverage that should only be used for finite periods (*n* = 55), (b) medications that involve a suggested trial period that is shorter than or unlisted by the BCPLC formulary (*n* = 15), (c) medications with fixed‐period BCPLC coverage that is longer than our reference duration of therapy (*n* = 8), (d) medications recommended for fixed‐period therapy, with lack of consensus on the best therapeutic duration (*n* = 13). Additionally, 28 medications considered potentially inappropriate by Beer's 2019 criteria exhibited DOTMs [8].

## 4 Discussion

BCPLC coverage criteria revealed inconsistencies that may contribute to inappropriate legacy prescribing and problematic polypharmacy, particularly with NSAIDs, PPIs, and bisphosphonates. Based on our findings, we recommend limiting NSAID coverage to a finite period with re‐evaluation for prolonged use; restricting PPI coverage to 8–12 weeks with renewals as needed; and replacing indefinite bisphosphonate coverage with an initial 5‐year period requiring subsequent renewals. These shorter coverage periods encourage regular reassessment, promoting safe, appropriate, and time‐limited medication use.

Although shorter coverage periods may promote safer, time‐limited prescribing, they also carry risks. In BC's ongoing primary care shortage, more frequent assessments and requests for prescription renewals could increase administrative burden, create access barriers for patients without a regular provider, and delay necessary treatment. A subset of patients might also require longer durations of NSAIDs, PPIs, or bisphosphonates based on clinical need. While these changes may shift workload toward primary care, frequent therapy re‐evaluation is standard‐of‐care, and renewals can be supported by other authorized providers, including community pharmacists or nurse practitioners. To prevent unintended harm, coverage policies that default to shorter intervals could include pharmacist‐initiated renewals under protocol, streamlined electronic reassessments, automated reminders with coverage grace periods, and clear criteria for extended coverage (e.g., PPI for Barrett's Esophagus). These measures could help balance appropriateness with access while minimizing avoidable treatment interruptions. Our findings align with evidence that drug coverage policy shapes prescribing. Subsidized patients often experience more polypharmacy, coverage changes can increase both low‐ and high‐value drug use, and expanded benefits can raise overall medication use [3, 4]. In British Columbia, limited coverage under BC Pharmacare may unintentionally encourage potentially harmful prescribing [6]. Unlike prior studies, we specifically examine policy design as a driver of suboptimal medication use, addressing a gap in understanding the unintended consequences of funding rules.

By systematically evaluating BCPLC criteria, we generated actionable recommendations supported by widely used clinical tools and regulatory guidance [5]. Limitations include the lack of data on actual prescribing patterns or patient outcomes, and a narrower resource pool than may capture all nuances. Future research should examine the impact of coverage policies on prescribing behavior and patient outcomes.

## 5 Conclusion

Policymakers in BC should reconsider indefinite coverage for medications with potential harm, including NSAIDs, PPIs, and bisphosphonates, to ensure therapy is regularly reassessed and to reduce the risk of problematic polypharmacy.

## Author Contributions

**Aydan Con:** data curation, formal analysis, investigation, methodology, project administration, writing – original draft, writing – review and editing. **Ivy Thrasher:** data curation, formal analysis, methodology, project administration. **Aaron M. Tejani:** conceptualization, data curation, formal analysis, methodology, project administration, supervision, validation, writing – original draft, writing – review and editing.

## Funding

The authors received no specific funding for this work.

## Conflicts of Interest

The authors declare no conflicts of interest.

## Transparency Statement

The corresponding author, Aaron M. Tejani, affirms that this manuscript is an honest, accurate, and transparent account of the study being reported; that no important aspects of the study have been omitted; and that any discrepancies from the study as planned (and, if relevant, registered) have been explained.

## Data Availability

The data that support the findings of this study are available on request from the corresponding author. The data are not publicly available due to privacy or ethical restrictions.
